# A novel model of acquired hydrocephalus for evaluation of neurosurgical treatments

**DOI:** 10.1186/s12987-021-00281-0

**Published:** 2021-11-08

**Authors:** James P. McAllister, Michael R. Talcott, Albert M. Isaacs, Sarah H. Zwick, Maria Garcia-Bonilla, Leandro Castaneyra-Ruiz, Alexis L. Hartman, Ryan N. Dilger, Stephen A. Fleming, Rebecca K. Golden, Diego M. Morales, Carolyn A. Harris, David D. Limbrick

**Affiliations:** 1 Department of Neurosurgery, Washington University in St. Louis School of Medicine, St. Louis, MO 63110 USA; 2 Division of Comparative Medicine, Washington University in St. Louis School of Medicine, St. Louis, MO 63110 USA; 3 Department of Surgery, Division of Neurosurgery, University of Calgary School of Medicine, Calgary, AB T2N 2T9 Canada; 4 Department of Animal Sciences, Division of Nutritional Sciences, Neuroscience Program, University of Illinois, Champagne-Urbana, Illinois, 61801 USA; 5 Traverse Science, Champaign, IL 61801 USA; 6 Department of Chemical Engineering and Materials Science, Wayne State University, Detroit, MI 48202 USA; 7 Department of Neurosurgery, Wayne State University School of Medicine, Detroit, MI 48202 USA; 8 Department of Pediatrics, St. Louis Children’s Hospital, St. Louis, MO 63110 USA; 9 Department of Neurosurgery, BJC Institute of Health, 425 S. Euclid, Campus, Box 8057, St. Louis, MO 63143 USA

**Keywords:** Hydrocephalus, Animal models, Kaolin, Acquired hydrocephalus, Shunt, Ventriculomegaly, Cognition

## Abstract

**Background:**

Many animal models have been used to study the pathophysiology of hydrocephalus; most of these have been rodent models whose lissencephalic cerebral cortex may not respond to ventriculomegaly in the same way as gyrencephalic species and whose size is not amenable to evaluation of clinically relevant neurosurgical treatments. Fewer models of hydrocephalus in gyrencephalic species have been used; thus, we have expanded upon a porcine model of hydrocephalus in juvenile pigs and used it to explore surgical treatment methods.

**Methods:**

Acquired hydrocephalus was induced in 33–41-day old pigs by percutaneous intracisternal injections of kaolin (n = 17). Controls consisted of sham saline-injected (n = 6) and intact (n = 4) animals. Magnetic resonance imaging (MRI) was employed to evaluate ventriculomegaly at 11–42 days post-kaolin and to plan the surgical implantation of ventriculoperitoneal shunts at 14–38-days post-kaolin. Behavioral and neurological status were assessed.

**Results:**

Bilateral ventriculomegaly occurred post-induction in all regions of the cerebral ventricles, with prominent CSF flow voids in the third ventricle, foramina of Monro, and cerebral aqueduct. Kaolin deposits formed a solid cast in the basal cisterns but the cisterna magna was patent. In 17 untreated hydrocephalic animals. Mean total ventricular volume was 8898 ± 5917 SD mm^3^ at 11–43 days of age, which was significantly larger than the baseline values of 2251 ± 194 SD mm^3^ for 6 sham controls aged 45–55 days, (p < 0.001). Past the post-induction recovery period, untreated pigs were asymptomatic despite exhibiting mild-moderate ventriculomegaly. Three out of 4 shunted animals showed a reduction in ventricular volume after 20–30 days of treatment, however some developed ataxia and lethargy, from putative shunt malfunction.

**Conclusions:**

Kaolin induction of acquired hydrocephalus in juvenile pigs produced an in vivo model that is highly translational, allowing systematic studies of the pathophysiology and clinical treatment of hydrocephalus.

**Supplementary Information:**

The online version contains supplementary material available at 10.1186/s12987-021-00281-0.

## Introduction

Hydrocephalus is a common neurological disorder in all ages [1–7] that is characterized by enlargement of the cerebral ventricles and often increased intracranial pressure. At present, treatment is limited to surgical diversion (shunt treatment) of cerebrospinal fluid (CSF) from the cerebral ventricles to alternative absorption sites or to endoscopic third ventriculostomy (ETV) with or without choroid plexus cauterization (CPC). Functional outcomes are problematic, with residual neurological and cognitive deficits prevalent in 25–80% of patients [1, 8–13].

A primary barrier to progress in improving treatments for hydrocephalus is a lack of large animal models of this disorder to elucidate the multifactorial pathophysiology of hydrocephalus and evaluate the effects of suboptimal surgical treatments [13, 14]. A major distinction involves the structural complexities of the brain, especially the differential responses to ventriculomegaly between lissencephalic and gyrencephalic species. Vink [15] has admirably reviewed this comparison in models of traumatic brain injury, noting the key biomechanical differences in gyrencephalic brains (porcine, canine, ovine, lagomorph, and non-human primate) that diffuse the pattern of pathology throughout the cerebral cortex and periventricular white matter as compared to lissencephalic species (mouse, rat). Most basic studies on the pathophysiology of hydrocephalus have used mouse and rat models, not large animals; the species differences have been implicated in the failure of many therapeutic studies in subsequent clinical trials, especially for traumatic brain injury [16, 17]. The lack of large animal models also impedes the development of improved shunting systems and novel surgical procedures, as well as pharmacological approaches designed to protect and/or repair the hydrocephalic brain. The need for large animal models of hydrocephalus has been recognized by the United States National Institutes of Health, who have sought to address this issue with *PA-18–623, “Tools to Enhance the Study of Prenatal and Pediatric Hydrocephalus”*.

Several gyrencephalic animal models of hydrocephalus have been utilized for experimental studies, including dogs [18–39], cats [40–59], ferrets [60–62], sheep [63–69], pigs [70, 71], and non-human primates [72–74]. Dogs and cats have been widely employed, especially in studies of CSF physiology and intracranial pressure; however, ethical concerns largely prevent their use today. Ferrets represent an excellent model of pre-term hydrocephalus due to the relative immaturity of their brains at birth [75], but they are too small to accommodate clinically-relevant shunt hardware or to perform novel techniques such as ETV ± CPC. Non-human primates have the advantage of being bipedal, but their use is cost prohibitive. Following a widely-used procedure in which the inert mineral kaolin is injected into the cisterna magna, fetal lambs develop hydrocephalus [65, 68, 76]. Johnston and colleagues expanded this model in adult sheep and provided preliminary observations on cytopathology and catheter obstruction following ventriculoperitoneal shunting. Unfortunately, sheep present several post-operative veterinary challenges, not the least of which is managing the potential exposure to biohazards, including Coxiella burnetti (Q-fever) [77]. Neonatal and adult pigs have also been used to model hydrocephalus following aqueductal stenosis [78] and intraventricular hemorrhage [70, 79–81]. The latter have provided valuable information on the pathogenesis and pathophysiology of post-hemorrhagic hydrocephalus. Except for preliminary attempts to reduce blood clot formation with recombinant tissue plasminogen activator delivered through an external ventricular drain [70, 81], these porcine studies did not include surgical shunting.

The domestic pig (*Sus scrofa*) is a preferred pre-clinical model because of its anatomic and physiologic similarities to humans, i.e. gyral patterns, 60:40 white:gray matter ratios [82–84], as well as similar brain growth and development timelines (maximum brain growth is late prenatal to early postnatal in both domestic pigs and humans) [83–87]. Advanced neuroimaging is often conducted on pigs [87–91], and the size of a pig brain [87] permits CSF shunting with clinical hardware. Juvenile pigs are amenable to the induction of hydrocephalus via intracisternal injections of kaolin or intraventricular injections of blood [70, 79–81, 92, 93]. Furthermore, stereotactic coordinates of the pig brain are well known [89, 94, 95]. Finally, cognitive testing is now available through the development of novel object recognition techniques in maturing pigs [96, 97].

To advance clinically relevant studies on the pathophysiology and treatment of hydrocephalus, we have developed a large animal model of juvenile hydrocephalus, induced with intracisternal kaolin injections, that can be used to test surgical treatments with clinical instrumentation. This report describes the methodology of this model and the initial observations on neuroimaging, ventriculomegaly, surgical treatment with ventriculoperitoneal shunting, and cognitive testing.

## Materials and methods

### Experimental design

Four experimental groups were studied in juvenile pigs (Table 1): (1) Untreated Hydrocephalus–animals that developed ventriculomegaly following intracisternal kaolin injections (n = 9); (2) Shunt-treated Hydrocephalus–animals with ventriculomegaly that received ventriculoperitoneal (VP) shunts for diversion of CSF (n = 8); (3) Sham Controls–animals that received intracisternal saline injections (n = 6); and (4) Intact Controls–normal naïve animals, only used for pre-induction MRI (n = 4). All groups were age-matched. Outcomes included pre- and post-induction behavior, neurological status, cognitive testing, neuroimaging with T1- and T2-weighted MRI, ventricular volume quantification from T2-weighted MRI scans, and gross brain morphology including subjective assessments of ventricular catheter patency; e.g., inspection of the catheter lumen for tissue or debris. In addition to the Untreated Hydrocephalus cases described above, 31 kaolin-injected untreated pigs were evaluated with MRI to subjectively assess the success rate of kaolin induction.

**Table 1:** Summary of cases

Case #	Condition	Age at induction (days)	Age at post-induction MRI (days)	Age at shunt treatment (days)	Age at Post-shunt MRI (days)	Survival age (days)	Duration Post-kaolin or post-shunt (days)	Total ventricular volume (mm^3^)*
1	UH	35	78			85	43	3310
2	UH	35	76			85	41	3350
3	UH	35	64			70	29	3027
4	UH	35	58			70	23	3592
5	UH	33	69			69	36	16,145
6	UH	33	69			69	36	12,802
7	UH	35	77			77	42	4391
8	UH	36	56			55	20	4601
9	UH	33	47			81	14	20,824
10	SH	37	48	53	81	81	28	3805/34127
11	SH	35	52	70	NA	80	10	3899/NA
12	SH	33	45	49	79	79	20	10,496/30533
13	SH	39	57	62	92	78	16	11,051/9035
14	SH	34	72	79	NA	84	5	9194/NA
15	SH	36	58	64	NA	94	30	12,710/NA
16	SH	36	58	63	NA	94	31	21,778/NA
17	SH	41	52	55	86	110	55	9554/6503
18	SC	44	53			83		2248
19	SC	44	45			83		2226
20	SC	46	54			93		2314
21	SC	46	55			93		2241
22	SC	45	51			82		2571
23	SC	42	51			82		1906
24	IC		34			21		2015
25	IC		41			89		2230
26	IC		37			54		2493
27	IC		37			72		1793
Misc	UH	28–35	38–82			38–82	10–51	Not used

*In cases with 2 ventricular volumes, the first number is the pre-shunt (untreated) condition and the second is the post-shunt condition

All procedures were approved by the Washington University Institutional Animal Care and Use Committee and done in an American Association for Accreditation of Laboratory Animal Care (AAALAC)-accredited facility in compliance with the Guide for the Care and Use of Laboratory Animals and the Animal Welfare Act. Juvenile (∼30-day old) domestic pigs (*Sus scrofa domesticus*) were obtained from a university approved vendor (Oak Hill Genetics LLC, Ewing, IL) and maintained in standard pens with raised flooring, fed a standard pig chow (Purina Porcine Grower Diet 5084) with ad lib access to water. Pigs were allowed to acclimate to the facility and physical exams were performed by institutional veterinary staff to assure normal health and behavior. Our study corresponds to the USDA Class D category of animal use. All animals undergoing surgery received surgical pre-anesthetics and analgesics, intra-operative anesthetics, and post-operative medications as needed (Additional file 5: Table 1).

### Induction of hydrocephalus

Pigs were sedated with a cocktail of telazol, ketamine, and xylazine (1.0 ml/50 lb intramuscular), intubated, and maintained under general anesthesia using 1–4% Isoflurane. The dorsal neck and head were shaved free of hair and the area was surgically prepped using povidone iodine and alcohol after the pig was positioned in a lateral decubitus position. Importantly, the head was temporarily flexed to about 90-degrees to open the atlanto-occipital interval as much as possible, with care taken to maintain a patent airway. Using sterile technique, a well-accepted method was employed to induce hydrocephalus [45, 51–53]: the cisterna magna was tapped percutaneously with an 18-gauge spinal needle (Becton-Dickenson 405184, McKesson Medical-Surgical. Richmond, VA) inserted in the midline at a 45° angle to the skin of the dorsal neck. The cisterna magna was located by manually sensing the needle tip as it contacted the flexible atlanto-occipital membrane and dura; if the occipital bone was encountered during penetration, the needle was “walked” inferiorly without withdrawing until the atlanto-occipital membrane was encountered. Accurate placement was confirmed when CSF was seen in the hub of the needle when the needle stylet was withdrawn. CSF was allowed to drip from the needle hub to confirm placement within the cisterna magna and checked for blood contamination. An IV extension tubing (B. Braun, ET12SB) was connected to the spinal needle and 1.25 ml of sterile 25% kaolin was injected slowly (about 0.25 ml/min) into the cisterna magna. After a 1–2-min equilibration period to allow dispersion of the kaolin, the IV extension tubing was disconnected from the spinal needle, and continued accurate placement was confirmed by observing a small amount of CSF mixed with kaolin emerging from the needle hub. The needle was withdrawn and the animal allowed to recover from anesthesia using standard veterinary procedures. When the animal could stand, it was placed in a recovery pen for 24–48 h and subsequently returned to its home cage.

### Surgical treatment with ventriculoperitoneal shunts

Following induction of hydrocephalus, standard VP shunts were placed because they are used almost exclusively to treat pediatric patients with hydrocephalus. We used CSF-Flow Control Shunt Kits (low-pressure valve/reservoir, model 9003D, Medtronic Neurosurgery, Goleta, CA, USA). Shunts were placed at 53–79 days of age, depending on the progression of hydrocephalus (primarily ventriculomegaly severity and neurological status) using similar neurosurgical procedures performed on human patients and feline models [50, 52]. Animals were anesthetized as described previously for the kaolin inductions. A 3–5 cm skin incision was made in the midline dorsal neck over the nuchal crest. The attaching occipital tissue was blunt-dissected off the occipital bone, with minimal use of bipolar cautery to release the muscle attachments. The muscle and skin were retracted to expose the occipital bone ~ 1 cm lateral to the midline on either side. A unilateral burr hole (~ 2.5 cm diameter) was made with a drill, the underlying dura mater was preserved, and any bone bleeding was stopped with bone wax. The location of this burr hole was determined from a pre-shunt MRI; this was usually centered 8.0–10.0 mm lateral to the midline and 10.0–12.0 mm inferior to the nuchal crest. Once the dura was exposed, attention was turned to the lateral thorax and abdomen for placement of the distal catheter. Two small (~ 1 cm) skin incisions were made; one over the left lateral rib cage for passing the distal catheter and inserting the valve with its reservoir and the other over the left abdomen ~ 1 cm caudal to the lowest rib for insertion of the peritoneal end of the distal catheter. The abdominal incision was dissected bluntly until the peritoneal layer was encountered. Two Crile hemostats were used to grasp the peritoneal layer and a small incision ~ 0.25 cm was made to expose the peritoneal cavity. A #1 Penfield instrument was inserted to confirm the peritoneal cavity had been accessed. A tunneler (Medtronic Neurosurgery, Goleta, CA, USA) was passed subcutaneously from the incision over the rib cage to the abdominal wound. A standard shunt catheter, distal slit valves removed, was then passed through the tunneler and inserted into the peritoneal cavity to a depth of about 20 cm. The proximal end of the subcutaneous catheter was attached to the distal port of a shunt valve and secured with 3–0 silk ties. A second portion of shunt tubing was passed subcutaneously from the occipital region to the rib cage incision using the same tunneling technique. The distal end of this catheter was attached to the proximal end of a reservoir with a low-pressure valve and secured with 3–0 silk ties. The valve was then positioned within a subcutaneous pocket over the rib cage and secured to underlying muscle and fascia with absorbable sutures. Once the subcutaneous portion of the shunt system had been completed, attention returned to the occipital area. Within the occipital craniotomy, bipolar cautery was used to coagulate the dura mater, which was then incised in a cruciate fashion with a #11 scalpel blade. The ventricular catheter was inserted through the opening in the dura mater and advanced through the occipital cortex into the lateral ventricle to a depth of about 2.5–3.5 cm from the occipital skull surface, based on pre-shunt measurements calculated from the MR images. Placement of the ventricular catheter in the lateral ventricle was confirmed by the appearance of CSF in the extracranial portions of the catheter. The ventricular catheter was anchored to the skull with Nylon sutures into the pericranium with or without a plastic anchor secured to the skull with self-tapping screws (Titanium cranial fixation system, Medtronic Brain Therapies, Irvine, CA, USA). The abdominal wall and chest incisions were closed in layers in a standard fashion with absorbable sutures. The cranial incision was closed in a layered fashion with absorbable sutures used to approximate the dorsal cervical muscles and overlying fascia in continuity with the galea aponeurotica. The skin was closed with subcuticular absorbable sutures and external interrupted 3–0 ETHILON® nylon non-absorbable sutures. External sutures were removed when the skin had healed, approximately 10-days post-surgery.

### Post-operative and post-MRI monitoring

During the recovery period (i.e., until a sternal position and/or standing could be achieved), animals were monitored every 15 min and vital signs recorded. Subsequently, monitoring was conducted about every 4–8 h until the animals could nourish themselves and displayed no neurological symptoms or signs of pain and discomfort (i.e., about 1–2 days),. After this period of recovery, daily monitoring was conducted to assure normal recovery from anesthesia following the MRI scans and the surgical procedures (i.e., healing of incisions, tissue swelling, integrity of the distal catheter track). The animal’s neurological status (i.e., general locomotor and sensorimotor behavior, ataxia, balance, alertness, reflexes, and ability to eat and drink) was also monitored. Body temperature and clinical activity during recovery were monitored closely as lethargy and hyperthermia/fever were often observed after kaolin injections. Acute neurological and behavioral signs and symptoms were managed medically with Buprenorphine/Buprenex, Tylenol/acetaminophen suppository, Carprofen/Rimadyl, Dexamethasone, Keppra, and occasional alcohol baths for hyperthermia (Additional file 5: Table 1).

### Neuroimaging and ventricular volume analyses

Anatomic MRI imaging was used to guide surgical implantation of ventricular catheters and determine gross morphological changes in the brain, especially volumetric increases in the cerebral ventricles, the location of the kaolin deposits, and the position of ventricular catheters. When possible, neuroimaging was performed pre-induction, post-induction/pre-shunting, and post-shunt on a Siemens Prisma 3.0-Tesla MR scanner with a 60-cm clear bore diameter, a 20-channel head coil, an 80 mT/m gradient field, and a slew rate of 200 mT/ms. The pig was anesthetized as described for hydrocephalus induction and positioned supine in the magnet. Breathing was controlled with a ventilator, respiratory and heart rates were monitored every 15 min, and oxygen saturation was monitored continuously with a pulse oximeter (Nonin® 7500, Nonin Medical, Plymouth, MN) secured to the tail. Depending on brain size, 54–192 slices of T1- and T2-weighted images were collected with a 3D fast spin-echo sequence with an echo train length of 8, FOV 205 × 205 mm (256 × 256 voxels), and a voxel size of 0.8 mm. T1 and T2 MPRAGE scan time varied from 4–11 min (T1:TR 2300 ms, TE 2.36 ms, 2 averages; T2: TR 3200 ms, TE 409 ms, 2 averages). Slice thickness was 900 µm. Data were provided in DICOM format (Digital Imaging and Communications in Medicine, standard for medical neuroimaging data).

Ventricular volume segmentation was performed using the free open-source software programs ITK-SNAP version 3.6.0 (http://www.itksnap.org/pmwiki/pmwiki.php). In ITK-SNAP, semi-autonomous methods were then used to separately label the lateral, third, fourth, and olfactory ventricles in each slice of the sagittal, axial (horizontal), and coronal view planes. Ventricular volume (mm^3^) for each case was obtained using the Volumes and Statistics tool within the Segmentation menu.

### Behavioral and cognitive testing

Well-established and validated tests developed by Dilger and Fleming [96–99] were used to quantify cognitive function prior to and following hydrocephalus induction using novel object recognition (NOR) tasks. NOR can reliably identify cognitive differences in infant pigs and is sensitive to small differences between treatment groups, as exemplified in a recent trial of dietary milk supplementation by Fleming et al. [98]. Using an arena designed from a standard housing pen with opaque walls and door and various objects secured to the floor, pigs were given two 10-min habituation periods over 2 days to explore the empty arena. The following day, they were then given 5 min to become familiar with 2 identical objects (sample trial). After a 24-h delay period, they were then given 5 min to explore 1 familiar object from the sample trial and 1 novel object (test trial). Each trial was recorded with a video camera suspended over the arena. A single, unbiased (i.e., blinded to treatment group), trained observer annotated and scored the continuous recordings to make the following measurements: (1) the number of visits to novel/sample objects, (2) time investigating novel/sample objects, (3) mean length of time per visit to an object, (4) latency to the first object visit, and (5) latency to the last object visit. The primary outcome for cognition was the Recognition Index (RI), which was calculated as the proportion of time each pig spent actively investigating the novel object as a proportion of the total active exploration time of both objects during the test trial. RI significantly > 0.50 indicates novelty preference and thus recognition memory [96, 98].

### Euthanasia and brain fixation

At designated time points, animals were sedated and anesthetized with Isoflurane and heparin was administered IV to facilitate cardiac perfusion. The anesthetized animals were euthanized using intravenous sodium pentobarbital (120 mg/kg intraperitoneal) or isoflurane (5% inhalation) and following cardiac arrest the chest was opened. The descending thoracic aorta was clamped, the pericardium opened, the right atrium incised to allow intracardiac perfusion, first with Phosphate-Buffered Saline (PBS) or sterile saline delivered via a peristaltic pump (Cole Parmer Masterflex L/S Easy load II, Vernon Hills, IL, USA) through a blunt 13-gauge needle inserted into the left ventricle followed by 4% paraformaldehyde (Sigma-Aldrich, St. Louis, MO, USA) in PBS to complete fixation. Depending on the size of the pig, 5–7 l of each solution were needed for adequate brain fixation. The brain and shunt system were removed using gross dissection and stored in fixative for 48 h and subsequently in 70% ethyl alcohol.

### Data analysis

For ventricular volume comparisons between sham controls and kaolin-injected animals, when a Kruskal–Wallis test indicated non-normal distribution of data, an unpaired *t-*test with Welch’s correction was used for non-parametric analyses. The primary outcome of cognitive function (i.e., learning and memory) was the Recognition Index (RI), which when altered significantly indicates novelty preference and thus Recognition Memory (RM) [96]. The linear mixed effects model was used to evaluate RM; this model is specified with RI as the outcome variable and a random intercept for animal. All data were reported as mean + standard deviation (SD) with statistical significance < 0.05.

## Results

### Cases

Altogether, 27 cases (of the 58 total) contributed ventricular volume and survival data to this report (Table 1), as follows: (A) Untreated, kaolin-induced hydrocephalic pigs (n = 9) survived to euthanization at an age of 55–85 (median 70) days. (B) Shunt-treated hydrocephalic pigs (n = 8) received VP shunts at 53–79 days of age and survived until euthanization at 78–110 days of age (median 81). Amongst the group of shunted pigs, 8 contributed to the untreated ventricular volume analysis (i.e., pre-shunt) whereas only 4 contributed to the post-shunt ventricular volume analysis. Controls included 6 sham/saline-injected pigs that survived 82–93 (median 83) days and 4 intact/non-injected animals that underwent neuroimaging at the approximate age at which experimental animals received kaolin, i.e., 34–41 (median 37) days of age.

### Post-induction results—kaolin obstruction and ventriculomegaly

In addition to the 17 hydrocephalic cases used for the ventricular volume analysis, 31 kaolin-induced cases were also studied for other purposes (n = 48). Altogether, these results showed that 85.4% (41/48 cases) of kaolin injections produced ventriculomegaly.

**Fig. 1:**
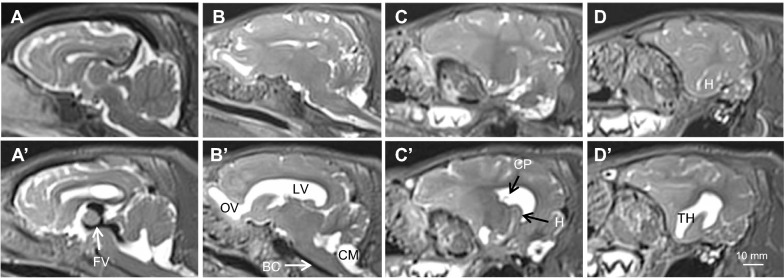
Representative T2-weighted sagittal MRI images summarizing brain and ventricular morphology in non-hydrocephalic control (**A**–**D**) and hydrocephalic kaolin-injected pre-shunt conditions (**A**′–**D**′); panels arranged left to right from midsagittal (**A**, **A**′) to lateral (**D**, **D**′). The intact control piglet (case 25) is 41-days old. Pre-shunt images are taken from case 13 at 18-days post-kaolin. In the pre-shunt condition, note the kaolin blockage of the basal cisterns (BC), prominent flow void (FV, black) within the third ventricle and cerebral aqueduct indicative of high CSF pulsatility, the patent channel connecting the olfactory ventricle (OV) to the lateral ventricle (LV), the choroid plexus (CP) floating in the LV, and the enlargement of all cerebral ventricles, especially the temporal horns (TH) containing the hippocampus (H), but no periventricular edema. The cisterna magna (CM) remains open. Scale bar = 10 mm for each all panels

**Fig. 2:**
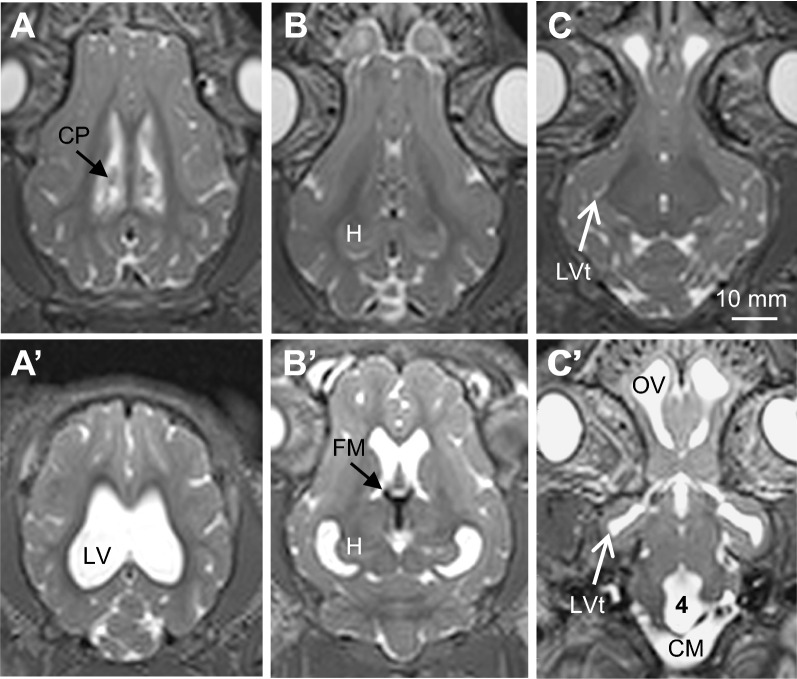
Representative T2-weighted axial MRI images summarizing brain and ventricular morphology in non-hydrocephalic control (**A**–**C**) and hydrocephalic pre-shunt (**A**′-**C**′) pigs. The intact control piglet (case 25) is 41-days old. Pre-shunt images are taken from case 13 at 18-days post-kaolin. In the pre-shunt condition, note the enlargement of all cerebral ventricles and the cisterna magna (CM), prominent flow voids (black) within the foramina of Monro (FM) and third ventricle indicative of high CSF pulsatility, the large olfactory ventricle (OV), and the choroid plexus (CP) in the lateral ventricle (LV). 4—fourth ventricle, H—hippocampus, LVt—temporal horn of the lateral ventricle. Scale bar = 10 mm for all panels

**Fig. 3:**
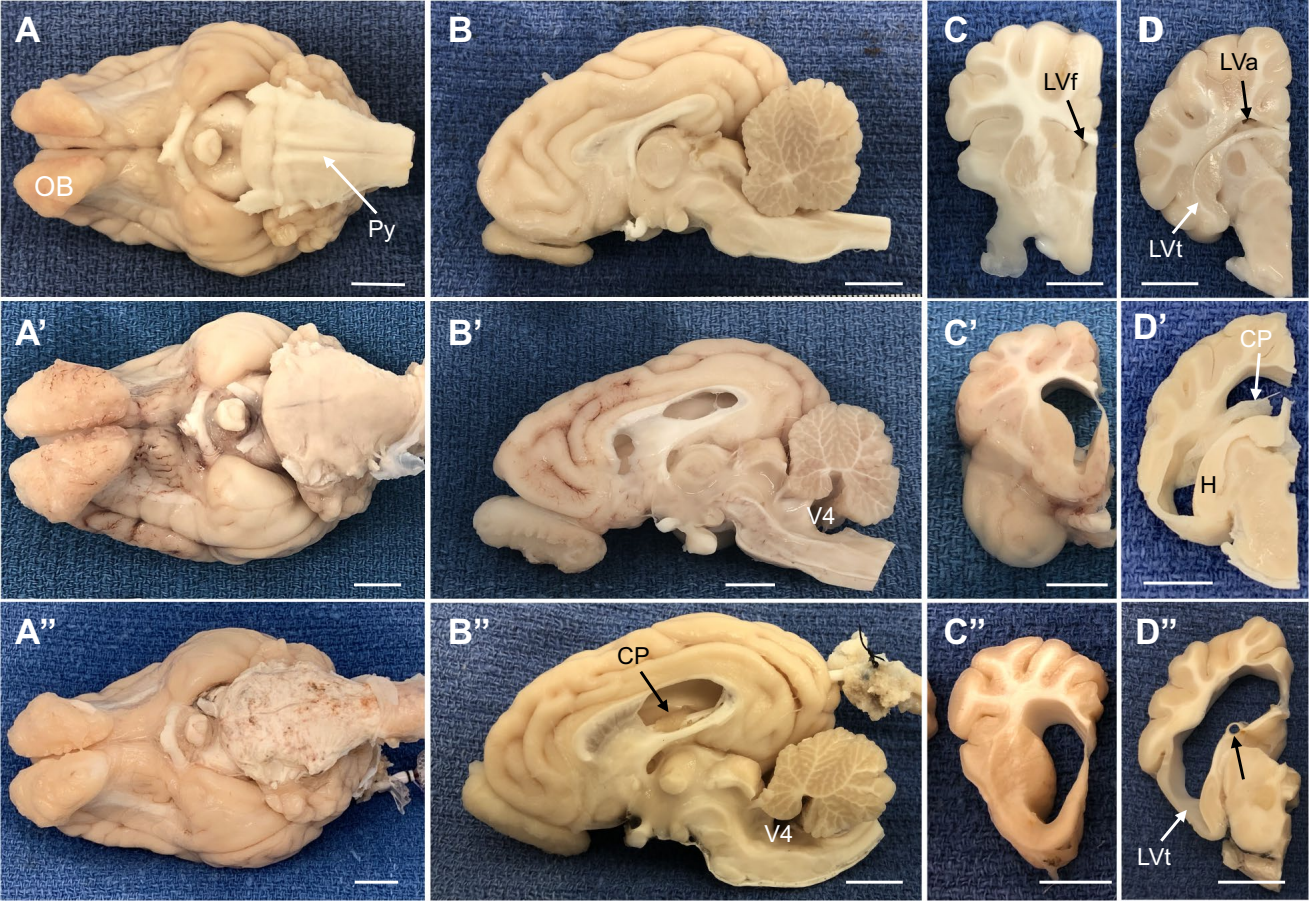
Representative gross morphological features of post-mortem non-hydrocephalic and untreated hydrocephalic pig brains (cases 21 and 9, respectively). In all cases, C and D columns contain coronal views from the same case as the midsagittal section in B. **A–D** In a sham control 93-day old animal, the myelinated pyramids (Py) were prominent and the atria (LVa), frontal horns (LVf), and temporal horns (LVt) of the lateral ventricle were small or slit-like. **A**′**–D**′ At 81-days old, kaolin deposits in the basal cisterns (BC) obscured the pyramids and ventriculomegaly was prominent throughout the ventricular system, including the 4th ventricle (V4). **A**″–**D**″. Shunted animals all retained the kaolin obstruction in the basal cisterns, and in some cases (this example), the ventricles were not reduced in size and the cortex lateral to the temporal horn (LVt) remained thin. The ventricular catheter contacted the choroid plexus (CP) but retained some open drainage holes, and a portion the catheter track (black arrow in D″) penetrated the fimbria. H—hippocampus. Scale bars–10 mm

In all kaolin-injected animals, at post-mortem examination, kaolin deposits consistently formed a solid cast within the basal cisterns (pontine, cerebellopontine angle, interpeduncular, and prepontine) and occasionally within the cistern of the lamina terminalis (Figures 1, 2, 3; Additional file 1: Figure S1, Additional file 2: Figure S2). Kaolin particles were rarely found within the cistern magna and never within the 4th- or 3rd-ventricles, the cerebral aqueduct, or the lateral ventricles. The basal cistern deposits could be identified on MRI throughout the post-induction and post-treatment survival periods on T2-weighted MRI scans (Figures 1B′ and 3A″) and were accompanied by arachnoiditis and adherence to the dura mater or membrane of Liliquist. In about half of the cases, thin, patchy kaolin deposits were found in the subarachnoid space surrounding the base of the hypothalamus and the pituitary gland. No kaolin deposits entered the parenchyma or any of the cerebral ventricles. The basilar artery and its branches were completely embedded in the kaolin cast.

Bilateral, symmetrical ventriculomegaly occurred in most cases. In 2 cases (<5), subsequent kaolin injections were needed to produce ventriculomegaly. All portions of the cerebral ventricles expanded, but no periventricular edema developed (Figures 1–3; Additional file 1: Figure S1, Additional file 2: Figure S2). It is noteworthy that olfactory ventricles were present normally in the domestic pig brain, each with a narrow channel connected to the frontal horn of each lateral ventricle; both the olfactory ventricle and the connecting channel expanded post-kaolin. The cisterna magna remained open and enlarged, with a thin membranous anterior wall that appeared to separate it from the 4th ventricle. Prominent CSF flow voids, indicative of high pulsatility, were conspicuous in the foramina of Monro, 3rd ventricle, and anterior cerebral aqueduct. The cerebral hemispheres remained gyrencephalic with relatively little distortion of the sulci and gyri. In contrast, the inferior wall of the temporal lobe became extremely thin in response to the considerable expansion of the temporal horn of the lateral ventricle (Figures 1D′ and 3D″, and Additional file 2: Figure S2E).

**Fig. 4:**
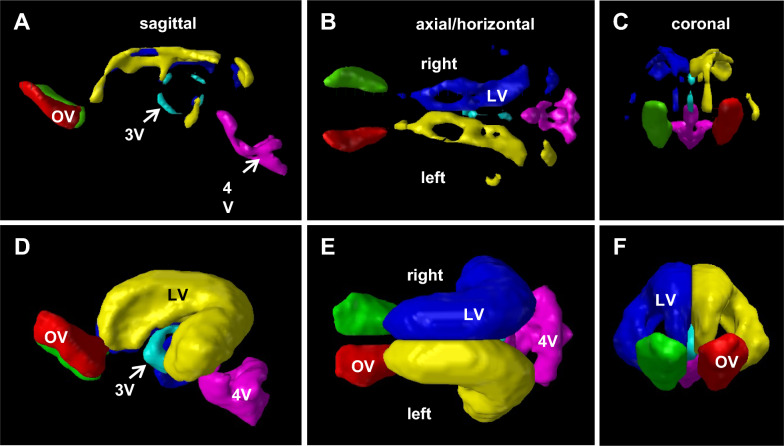
Ventricular volume reconstructions of representative age-matched sham control (**A**–**C**) and untreated hydrocephalic pigs (**D**–**E**). Note the CSF-filled olfactory ventricles (OV) in normal animals and the dramatic expansion of all cerebral ventricles following intracisternal kaolin injections. Total ventricular volumes were 1906 mm^3^ and 21,778 mm^3^ for the sham (case 23) and untreated hydrocephalic (case 16, post-induction MRI), respectively

**Fig. 5:**
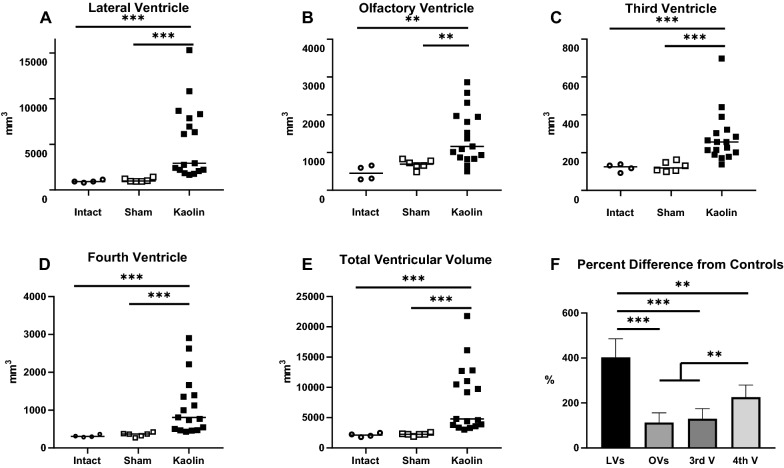
Ventricular volumes of intact (n = 4), sham-injected (n = 6), and kaolin-injected (n = 17) pigs. **A–E** Dot plots illustrating volumes for individual and combined ventricles. Note that the mean ventricular volume increased significantly in all ventricles in kaolin-injected animals compared to both intact and sham controls. No significant differences between sham and intact controls were found. The dashed lines in all ventricular volume graphs indicate 2.0 standard deviations from the sham control mean. This threshold was used to define the existence of hydrocephalus, such that all cases were considered hydrocephalic when total ventricular volumes were compared (**E**) **F** The percent difference in mean ventricular volume from control values confirmed that all ventricles expanded post-kaolin, and that proportionally the lateral ventricles increased in size significantly more than any of the other ventricles. **p < 0.01; ***p < 0.001

**Fig. 6:**
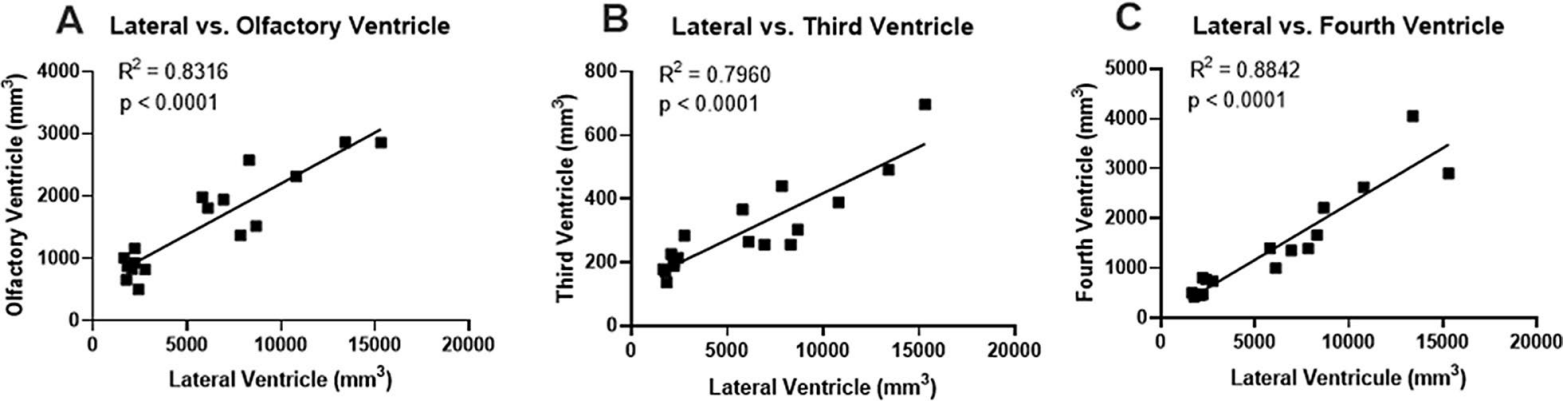
Ventricular volume correlations between the lateral ventricle and the olfactory, third, and fourth ventricles in the same pigs used previously for the untreated ventricular volume measurements (cases 1–17, post-induction MRI). All 3 comparisons were statistically significant (p < 0.001) with high R^2^ values

Volumetric assessments revealed that ventriculomegaly occurred post-induction in all cerebral ventricles (i.e., olfactory, lateral, 3rd, and 4th ventricles; Figs. 4–6). Quantification of ventricular volume confirmed the extent and variability of ventriculomegaly (Fig. 5). Ventricular volume increased significantly (p < 0.001) in all ventricles compared to both intact and sham controls. Regional variations existed within the cerebral ventricles; proportionally, the lateral ventricles expanded the most, followed by the 4th ventricle. All 17 untreated cases (no-shunt or pre-shunt) exhibited lateral and total ventricular volumes that were above 2 standard deviations from the sham control mean. We arbitrarily set this threshold to determine which cases were “hydrocephalic”. Linear regression revealed highly significant correlations between lateral ventricular volume and olfactory, third, and fourth ventricle volumes (Fig. 6).

### Post-induction neurological and cognitive outcomes

For about 24-hours following kaolin injections and recovery, pigs were mildly lethargic with most of the cases exhibiting increases in body temperature (103.5–108.0 °C; normal = 100.5–103.5 °C) and some showed mild ataxia (imbalance, wider stance, stretching of hindlimbs). When this acute response occurred, it usually was resolved within 2–3 days with appropriate medical management (Buprenorphine/Buprenex, Tylenol suppository, Carprofen/Rimadyl, Baytril, and Dexamethasone) and with occasional alcohol baths for hyperthermia. Past 2–3 days post-kaolin, these animals did not exhibit any signs of pain or discomfort, and were alert with normal reflexes, consumed food and water, and gained weight throughout the post-kaolin period. Nevertheless, neuroimaging revealed ventriculomegaly in most of these animals (see Additional file 3: Figure S3).

Preliminary results from cognitive testing (Additional file 4: Figure S4 showed a non-significant trend toward a higher Recognition Index and significantly more exploratory time (p = 0.008) in the post-induction animals compared to normal (pre-induction) pigs, suggesting possible learning impairment; i.e., more time was needed to become familiar with the novel object.

### Post-shunt results—catheter placement and ventriculomegaly

**Fig. 7:**
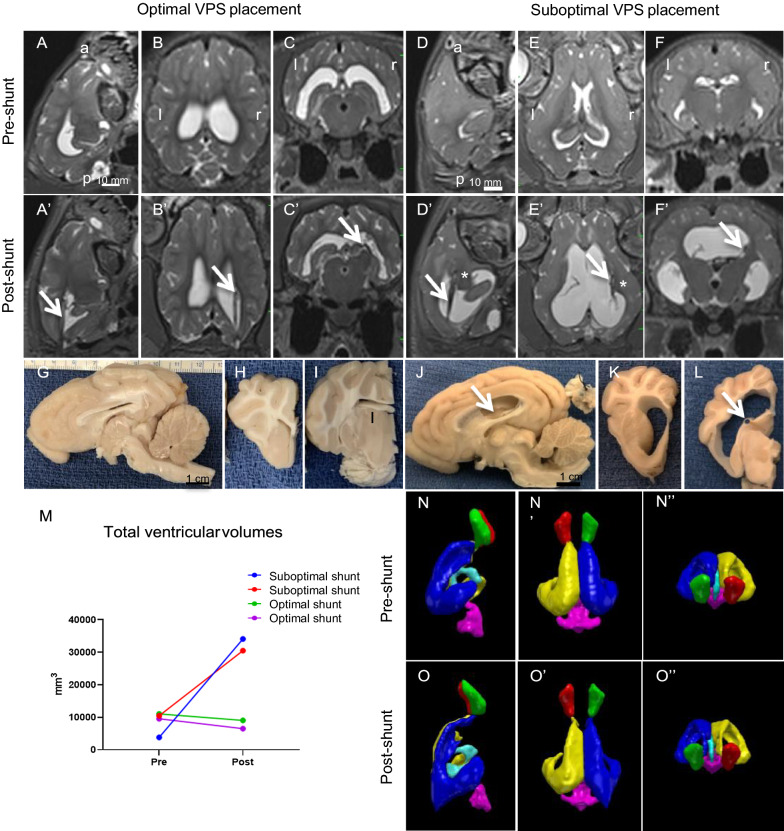
Representative optimal and sub-optimal shunt placements in hydrocephalic pigs after 20–30 days of treatment. **A–F**′. T2-weighted sagittal, axial, and coronal MRI images illustrating optimal (case 13, **A**–**C**′) and sub-optimal (case 10 **D**–**F**′) shunt placement. Pre- and post-shunt images were taken from the same two pigs. Shunting reduced (but did not eliminate) the flow void in the third ventricle and decreased the size of all ventricles when optimally placed, but not to control levels. In the optimal case, the arrows indicate the catheter (**A**′–**C**′) inside of the right lateral ventricle. In the sub-optimal case (**D**′–**F**′), arrows indicate the catheter and asterisks indicate the catheter tip (**D**′–**F**′). **G**–**L** Representative gross morphological features of shunt-treated hydrocephalic pig brains (Case 13 for optimal placement, and Case 12 for sub-optimal case). In sub-optimal case, the catheter (arrow in **J**) was located along the dorsolateral wall of the lateral ventricle with some contact with the choroid plexus. In all cases, **H** and **I**, **K** and **L** columns contain coronal views from the same case as the midsagittal sections in **G** and **J**, respectively. In the optimally placed shunt case, the ventricles were reduced in size but not in the sub-optimal placement. In **J** and **L**, the ventricular catheter contacted the choroid plexus (CP) but retained some open drainage holes, and the catheter track (black arrow in **J** and **L**) contacted the CP. **M** Graph showing the total ventricular volumes in mm^3^ before (pre) and after (post) 20–30 days of shunt treatment (Sub-optimal cases are case 10 -blue- and case 12-red-; optimal cases are 13-green- and 17 –purple-). **N**–**P** Ventricular volume reconstructions of a representative optimal shunt placement before and after shunt treatment in the same cases used for the MRI (case 13). Note the reduction of the total ventricular volume after 30 days of shunt treatment. Abbreviations: a, anterior, l, left, p, posterior, r, right. Scale bars = 10 mm for all the MRI scan and gross anatomy panels

Although neuroimaging was not performed on all 8 shunted cases due to logistics, available post-shunt scans revealed optimal (n = 2) and sub-optimal (n = 2) shunt placements. In cases 13 and 17 with optimal shunt placement, ventriculomegaly was reduced (Fig. 7A–C. In the suboptimal shunt cases 10 and 12 the proximal portion of the ventricular catheter was deeply embedded in periventricular tissue (Fig 7D′–F″) and ventriculomegaly increased.. In pilot studies, while attempting different trajectories (including coronal), gross anatomy revealed that the tip of the catheter occasionally penetrated the head of the caudate nucleus, the thalamus, the hippocampus, or the periventricular white matter. Subsequently, when the insertion of the catheter was limited to 3.5 cm from the external surface of the occipital skull to the approximate location of the foramen of Monro, more consistent and optimal placement within the lateral ventricle was achieved (Fig. 7), with the drainage holes open to CSF. In many cases, at least a portion of the catheter contacted the ventricular wall or the choroid plexus (sub-optimal placement, Fig. 7). Contact with the choroid plexus was occasionally associated with hemorrhage throughout this structure, and in these cases, blood was present in the distal valve and catheter. On two occasions, the plastic anchor, which had been sutured to the occipital bone and the catheter as it exited the skull, had become detached. This detachment allowed the ventricular catheter to migrate superficially, in one case dorsally into the parietal and occipital cerebral cortex; in the other case, the catheter had exited the cranial cavity and the proximal tip was located within the cervical musculature. Importantly, because both of these sub-optimal cases were asymptomatic for the entire 30-day post-shunt survival period, it is likely that CSF drainage had been effective for at least a portion of the treatment period. We now use Stealth guidance to optimize the trajectory to the lateral ventricle.

### Post-shunt complications

The most prevalent complication was sub-optimal placement of the ventricular catheter, especially in the initial cases. It was difficult to sense tissue penetration as the catheter was advanced (sub-optimal shunt placement, Fig. 7); therefore, it was necessary to rely on the pre-shunt MRI for trajectory planning. As we gained experience, several parameters became very important for optimal placement of the ventricular catheter: (1) The dorsoventral ‘height’ of the lateral ventricle should be at least 6.0 mm for a standard catheter with an outside diameter of 2.3 mm; we were most successful when this dimension was 10–13 mm. (2) Insertion should be no more than 3.5 cm from the surface of the occipital bone; this placed the proximal tip of the catheter near the foramen of Monro and prevented penetration of the caudate nucleus. (3) Insertion should be parallel to the dorsal surface of the skull; the tendency to advance perpendicular to the face of the occipital bone caused the catheter to be angled inferiorly with penetration of the thalamus, caudate nucleus, and/or hippocampus. (4) A plastic anchor, screwed into the occipital bone, should be used to secure the ventricular catheter in place; the Medtronic anchor supplied with standard catheters was ideal for this purpose.

Post-shunt periods less than 28 days were characterized by relatively acute onset of neurological symptoms (n = 4/8, cases 11–14); often demise began 6–24 h before euthanasia was required, and in two cases animals that appeared normal developed profound seizures just 8–10 h later. Shunt patency studies were not performed in vivo, so it is not possible to know unequivocally if these cases represented shunt malfunction. No shunted animals became febrile or developed infections.

## Discussion

This report demonstrates that percutaneous injections of kaolin frequently produce significant moderate to severe bilateral ventriculomegaly in all cerebral ventricles, including the olfactory ventricle, which in pigs is patent normally but expands further during hydrocephalus. Conspicuous CSF flow voids were also observed in the third ventricle, foramina of Monro, and cerebral aqueduct. Kaolin deposits were primarily confined to the basal cisterns where they formed a solid cast that encased the subarachnoid arteries and veins; although kaolin injections were targeted to the cisterna magna and clear CSF was obtained prior to injection, this portion of the subarachnoid space was patent. Because pigs do not have a foramen of Magendie [100, 101] ; kaolin within the cerebellopontine angle may block the foramina of Luschka and be sufficient to cause marked enlargement of the 4th ventricle. Most untreated pigs with ventriculomegaly remained asymptomatic for relatively long post-induction periods, although preliminary novel object recognition tests revealed cognitive changes. Ventriculoperitoneal shunting was possible, with complications suggestive of shunt failure occurring in about half of these cases.

The development of this model builds on previous work in several gyrencephalic animals, some of which represent maturing infantile and juvenile brains. Many studies of normal and altered CSF physiology and ICP have been performed on felines, canines, and non-human primates, and the data produced by these experiments have provided the foundation for the current understanding of CSF and ICP biomechanics. More recent experiments in hydrocephalic kittens and dogs have revealed the cytopathology commonly produced by ventriculomegaly [23, 44, 45, 49–51, 53, 57, 58, 102, 103] and the importance of CSF pulsatility [104–108]. Continued studies on these species have been thwarted by societal concerns for the well-being of these animals, and many institutions are reluctant to permit chronic survival experiments in these hydrocephalic models; the studies that have continued have become highly regulated and expensive. Nevertheless, FDA approval of new therapeutic devices and sensors often requires pre-clinical proof of biocompatibility and efficacy in animal models that are large enough to accommodate clinical devices; this is especially true for novel approaches that seek to improve shunt technology. The need for more translational in vivo models has been recognized by the NIH in their small business incentive programs and promoted by the Hydrocephalus Association, which helped produce an important white paper emphasizing the inclusion of large animal models in experimental studies [13, 14, 109]. Importantly, because of the chronic nature of hydrocephalus and the need for long-term treatments, this model needs further development in terms of the growth of these animals (i.e., the need to lengthen the distal catheter), progressive ventriculomegaly in untreated animals, and the impact of repetitive shunt malfunctions.

It is worth noting that porcine species normally have a somewhat unique CSF-filled chamber within large olfactory bulbs that communicate with the lateral ventricles. Absorption of CSF into the nasal lymphatics that surround the olfactory nerves as they pass through the cribriform plate was initially discovered in rabbits, cats, and non-human primates by McComb and his colleagues in 1975 [110–112]. More recently, Johnston et al. have shown conclusively that this CSF outflow pathway exists in a variety of species, including humans, and have promoted the notion that nasal lymphatics could at least provide an alternative CSF absorption route when other pathways are obstructed [113–119]. This pathway could conceivably be a functional mechanism for relieving CSF pressure in our pig model, thereby allowing long symptom-free durations.

Post-induction ventriculomegaly was variable in our cases and did not appear to be correlated with the extent of kaolin obstruction or the post-kaolin duration. However, there was a strong correlation between lateral ventricle and olfactory ventricle size. While the variation in ventricular volume amongst cases highlights the need to use neuroimaging to confirm ventriculomegaly, it is worth noting that similar variations occur clinically in patients with hydrocephalus and that ventriculomegaly is not predictive of outcome unless it is quite severe [120–124]. To define “hydrocephalus” consistently, we applied thresholds of 1.0 and 2.0 standard deviations from the sham control mean ventricular volumes to the post-kaolin cases. This approach demonstrated that all cases were considered “hydrocephalic” when the mean lateral ventricular volumes and the combined lateral and olfactory ventricle volume were above 2.0 standard deviations from controls. We recommend that this approach be used in other experimental studies to standardize different types of hydrocephalus [125]. Unfortunately, we did not have enough intermediate MRI time points during the post-kaolin to pre-shunt period to show age-related progressive changes in ventriculomegaly.

Neurological deficits and signs of pain or discomfort were primarily confined to the immediate post-induction period and at the onset of shunt malfunction. In vivo neuroimaging and gross morphological observations confirmed that the intracisternal kaolin injections consistently produced large casts that completely blocked the basal cisterns. Increased body temperature almost always occurred 3–24 h post-kaolin, and while it could be controlled with medications such as Tylenol, Rimadyl (Carprofen), and Dexamethasone, it seemed highly predictive of a successful kaolin injection. Other behavioral and neurological signs, such as mild ataxia, lethargy, loss of appetite, head tilt, and coarse hair coat abated within 1–2 days post-induction, and most animals remained symptom-free for 1–4 weeks. These signs and symptoms returned, however, in 4 of 8 shunted animals within 5–20 days post-shunt. Usually, these behavioral changes were gradual enough to allow neuroimaging and collection of tissue before the shunt malfunction caused further demise, but it was not uncommon for rapid deterioration to occur. The contrast between untreated animals with no signs or symptoms for 14–43 days post-kaolin and these shunted cases suggests that shunt malfunctions may have been responsible for the early demise; an alternative possibility is that animals with the largest ventricles were chosen for shunt treatments to optimize the feasibility of the surgery.

The ability to reduce ventriculomegaly with shunting in an in vivo model is encouraging. Although further improvements could be made for optimal and consistent placement of the ventricular catheter, most notably with the aid of neuronavigation, shunting can be performed successfully with clinical drainage systems. It is worth noting that our occipital approach placed the ventricular catheter close to the choroid plexus, an issue which has prompted many neurosurgeons to favor a frontal approach in the treatment of children. We are currently exploring a frontal approach in which the ventricular catheter is inserted orthogonally into the frontal horn of the lateral ventricle. Access to a distal reservoir also allows serial taps for CSF samples for subjective assessments of shunt function and evaluation of CSF biomarkers. In addition to permitting experimental studies of the effects of shunt timing on pathophysiology, this model allows systematic evaluations of novel shunt designs. For example, several of our ongoing studies with this model involve biocompatibility (i.e., safety) evaluations, efficacy of new catheter materials and designs, and tests of CSF flow detection monitors. Intraoperative guidance, when available, should improve the placement of ventricular shunt catheters. Furthermore, our preliminary experience with neuroendoscopic procedures, specifically ETV and ETV-CPC, demonstrates that these treatment options can be explored systematically and produce promising results in this model. A study detailing ETV and ETV-CPC in this model is forthcoming.

In Conclusion, kaolin injections into the cisternal magna of juvenile pigs produced significant bilateral ventriculomegaly in all regions of the cerebral ventricles in approximately 85% of cases, with kaolin deposits and arachnoiditis forming a substantial obstruction in the basal cisterns. The cisterna magna was patent and enlarged and a thin membrane separated it from the posterior wall of the 4th ventricle. A few animals required a second kaolin injection to produce ventriculomegaly. Ventricular volume data suggested that a threshold of 2 standard deviations above the mean value for sham controls could be considered as the defining point for classifying cases as “hydrocephalic”. Untreated pigs were asymptomatic for up to 43 days post-induction. Commercially-available ventriculoperitoneal shunts were placed successfully in hydrocephalic animals. These shunts, if patent, remained functional for 30–50 days. However, if tissue and blood obstructed the ventricular catheter or the distal valve, shunt failure occurred at 3–21 days post-shunt. Preliminary cognitive tests designed specifically for pigs suggested that untreated hydrocephalic pigs spent more time exploring novel objects. This reliable model of acquired juvenile hydrocephalus is highly translational, allowing for systematic studies of the pathophysiology and clinical treatment of hydrocephalus.

## Supplementary Information

**Additional file 1: Fig.S1.** Representative T2-weighted MRI images summarizing brain and ventricular morphology in non-hydrocephalic control (**A-F**), hydrocephalic just prior to shunting (**A’-F’**), and post-shunt (**A”-F”**) piglets. The intact control piglet (case 25) is 41-days old. Pre- and post-shunt images are taken from the same piglet (case 13 at 18-days post-kaolin and 30-days post-shunt, respectively. (**A’-F’**) In the pre-shunt condition, note the prominent flow void (FV, black profile) within the third ventricle and cerebral aqueduct (**A’**) indicative of high CSF pulsatility, the black signal indicating kaolin blockage of the basal cisterns (BC in **B’**), the patent channel connecting the olfactory ventricle (OV) to the lateral ventricle (LV), the choroid plexus (CP) floating in the LV (**C’**), and the enlargement of all cerebral ventricles, especially the temporal horns (TH) containing the hippocampus (H). The cisterna magna (CM) remains open. (**A”-F”**) Shunting reduced (but did not eliminate) the flow void in the third ventricle (**A”**) and decreased the size of all ventricles, but not to normal levels. In this case, the catheter (D”, arrows indicate brain entry and the catheter located along the dorsal wall of the LV with some contact with the choroid plexus. Scale bars = 10 mm for all panels.

**Additional file 2: Fig. S2.** Representative T2-weighted coronal images summarizing brain and ventricular morphology in non-hydrocephalic control (A-J) and hydrocephalic pre-shunt (A’-J’) pigs; panels arranged left to right from anterior to posterior and matched to corresponding levels (i.e. A and A’). Pre-shunt images are taken from case 13 at 18-days post-kaolin. The control pig (case 25) is 41-days old. In the hydrocephalic pre-shunt condition, note the enlargement of all cerebral ventricles and the cisterna magna (CM), prominent flow voids (black) within the third ventricle (3, E’) indicative of high CSF pulsatility, the large olfactory ventricles (OV), and the choroid plexus (CP) in the lateral ventricle LV, and the kaolin (black) blockage of the basal cisterns (BC) and foramina of Luschka (FL). 4 – fourth ventricle, H – hippocampus, FH – frontal horn, FM – foramen of Monro, OH – occipital horn, TH – temporal horn. Scale bar = 10 mm for all panels.

**Additional file 3: Fig.S3.** Representative T2-weighted axial MRI images summarizing brain and ventricular morphology in non-hydrocephalic control (A-E) and hydrocephalic just prior to shunting (A’-E’) pigs. The control pig (case 25) is 41-days old. Pre- shunt images are taken from pig case 13 at 18-days post-kaolin. In the pre-shunt condition, note the enlargement of all cerebral ventricles and the cisterna magna (CM), prominent flow voids (black) within the foramina of Monro (in C’) and third ventricle/cerebral aqueduct (in C’ and D’) indicative of high CSF pulsatility, the patent channel connecting the olfactory ventricle (OV) to the lateral ventricle (LV), and the choroid plexus (CP) floating in the LV. LVt – temporal horn of the lateral ventricle. Scale bar = 10 mm for all panels.

**Additional file 4: Fig.S4.** Real-time video segment of a typical cognitive test. Initially, during the habituation phases, the same 2 objects are available for the pig to explore. After a rest day, the same pig is tested again with one of the previous “familiar” objects (the rectangular pan) and a novel object (the round pan). The time spent exploring each object is recorded. In this case, the animal spent more time exploring the novel object, indicating that it has remembered the familiar object. Cognitively impaired animals will spend disproportionately more time exploring the initial, presumably familiar, object [96, 97].

**Additional file 5: Table S1.** Medications.

## Data Availability

All data generated or analyzed during this study are included in this published article and its Additional files.
