# Supplementary figures and images for: A novel model of acquired hydrocephalus for evaluation of neurosurgical treatments
Source: Fluids Barriers CNS. 2021 Nov 8;18:49. doi: 10.1186/s12987-021-00281-0 (PMC8576945; doi:10.1186/s12987-021-00281-0)

## Slide 1
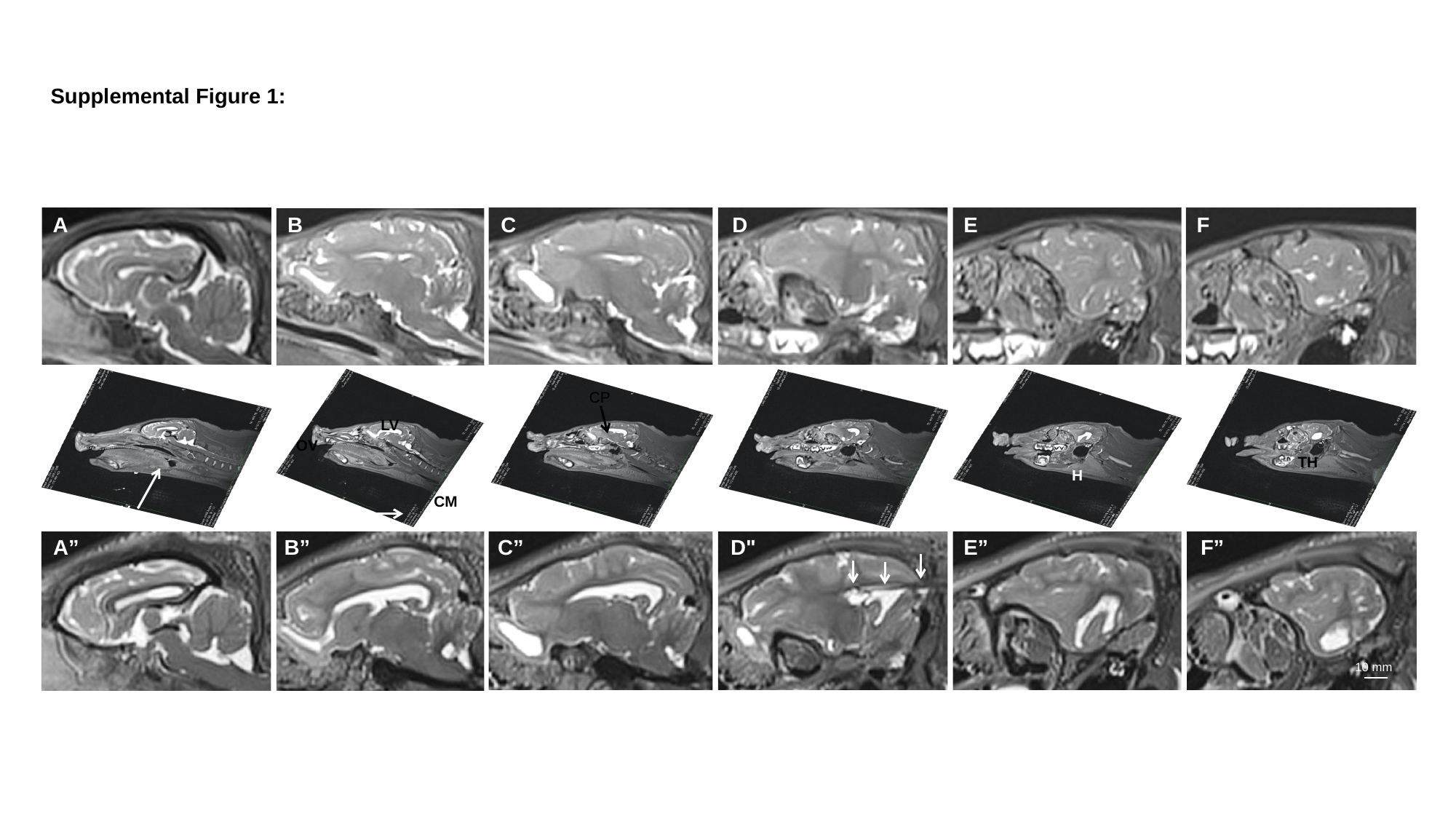

Supplemental Figure 1:
A
B
C
D
E
F
A’
B’
C’
D’
E’
F’
CP
LV
OV
TH
H
CM
FV
BC
A”
B”
C”
D"
E”
F”
10 mm

Supplement: Supplementary file 1 — Additional file 1: Fig.S1. Representative T2-weighted MRI images summarizing brain and ventricular morphology in non-hydrocephalic control (A-F), hydrocephalic just prior to shunting (A’-F’), and post-shunt (A”-F”) piglets. The intact control piglet (case 25) is 41-days old. Pre- and post-shunt images are taken from the same piglet (case 13 at 18-days post-kaolin and 30-days post-shunt, respectively. (A’-F’) In the pre-shunt condition, note the prominent flow void (FV, black profile) within the third ventricle and cerebral aqueduct (A’) indicative of high CSF pulsatility, the black signal indicating kaolin blockage of the basal cisterns (BC in B’), the patent channel connecting the olfactory ventricle (OV) to the lateral ventricle (LV), the choroid plexus (CP) floating in the LV (C’), and the enlargement of all cerebral ventricles, especially the temporal horns (TH) containing the hippocampus (H). The cisterna magna (CM) remains open. (A”-F”) Shunting reduced (but did not eliminate) the flow void in the third ventricle (A”) and decreased the size of all ventricles, but not to normal levels. In this case, the catheter (D”, arrows indicate brain entry and the catheter located along the dorsal wall of the LV with some contact with the choroid plexus. Scale bars = 10 mm for all panels. [file 12987_2021_281_MOESM1_ESM.pptx]

## Slide 1
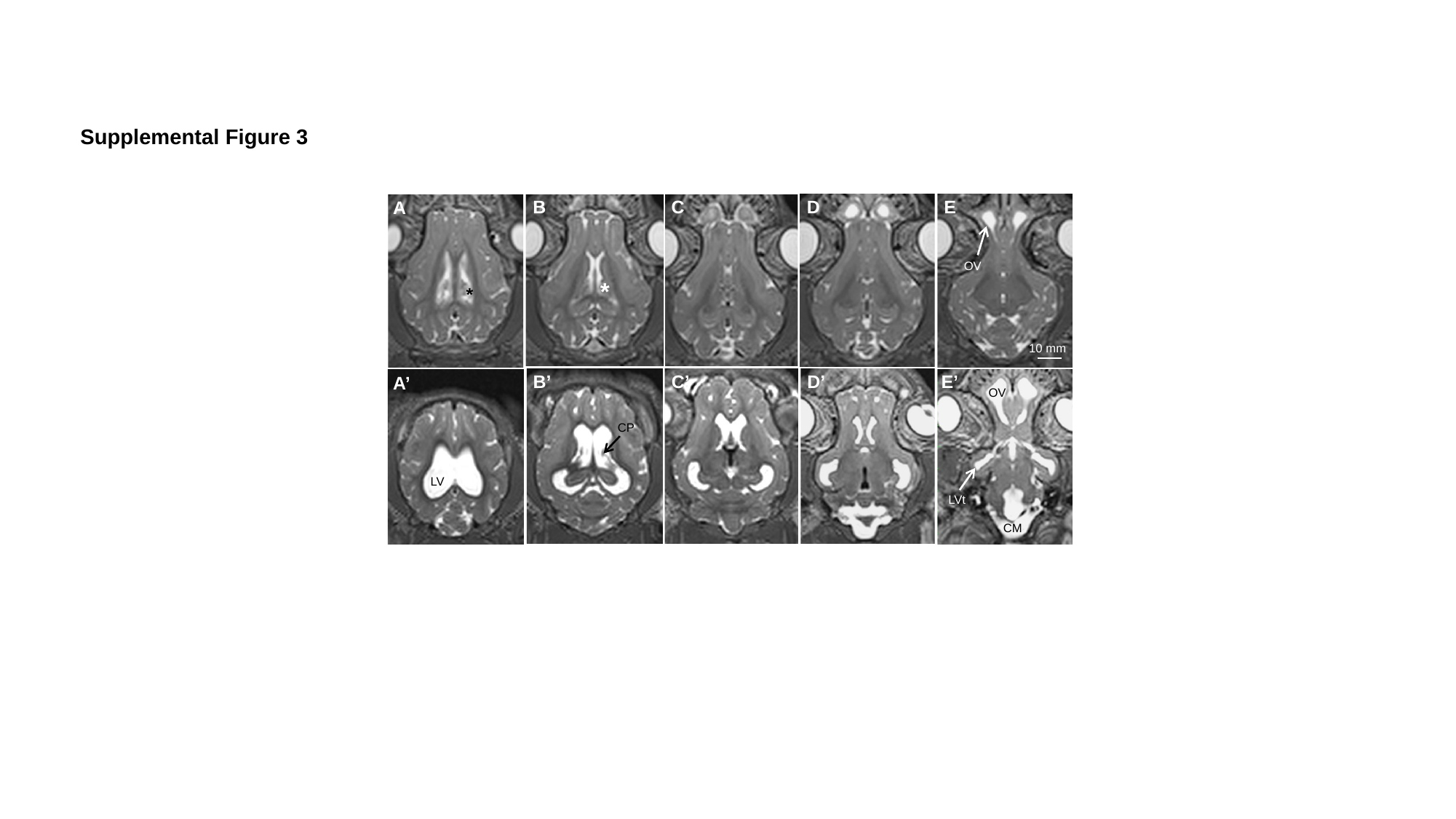

Supplemental Figure 3
C
D
E
B
A
OV
*
*
10 mm
B’
C’
D’
E’
A’
OV
CP
LV
LVt
CM

Supplement: Supplementary file 3 — Additional file 3: Fig.S3. Representative T2-weighted axial MRI images summarizing brain and ventricular morphology in non-hydrocephalic control (A-E) and hydrocephalic just prior to shunting (A’-E’) pigs. The control pig (case 25) is 41-days old. Pre- shunt images are taken from pig case 13 at 18-days post-kaolin. In the pre-shunt condition, note the enlargement of all cerebral ventricles and the cisterna magna (CM), prominent flow voids (black) within the foramina of Monro (in C’) and third ventricle/cerebral aqueduct (in C’ and D’) indicative of high CSF pulsatility, the patent channel connecting the olfactory ventricle (OV) to the lateral ventricle (LV), and the choroid plexus (CP) floating in the LV. LVt – temporal horn of the lateral ventricle. Scale bar = 10 mm for all panels. [file 12987_2021_281_MOESM3_ESM.pptx]
