# Supplementary material for: A novel model of acquired hydrocephalus for evaluation of neurosurgical treatments
Source: Fluids Barriers CNS. 2021 Nov 8;18:49. doi: 10.1186/s12987-021-00281-0 (PMC8576945; doi:10.1186/s12987-021-00281-0)
Supplement: Supplementary file 2 — Additional file 2: Fig. S2. Representative T2-weighted coronal images summarizing brain and ventricular morphology in non-hydrocephalic control (A-J) and hydrocephalic pre-shunt (A’-J’) pigs; panels arranged left to right from anterior to posterior and matched to corresponding levels (i.e. A and A’). Pre-shunt images are taken from case 13 at 18-days post-kaolin. The control pig (case 25) is 41-days old. In the hydrocephalic pre-shunt condition, note the enlargement of all cerebral ventricles and the cisterna magna (CM), prominent flow voids (black) within the third ventricle (3, E’) indicative of high CSF pulsatility, the large olfactory ventricles (OV), and the choroid plexus (CP) in the lateral ventricle LV, and the kaolin (black) blockage of the basal cisterns (BC) and foramina of Luschka (FL). 4 – fourth ventricle, H – hippocampus, FH – frontal horn, FM – foramen of Monro, OH – occipital horn, TH – temporal horn. Scale bar = 10 mm for all panels. [file 12987_2021_281_MOESM2_ESM.pptx]

## Slide 1
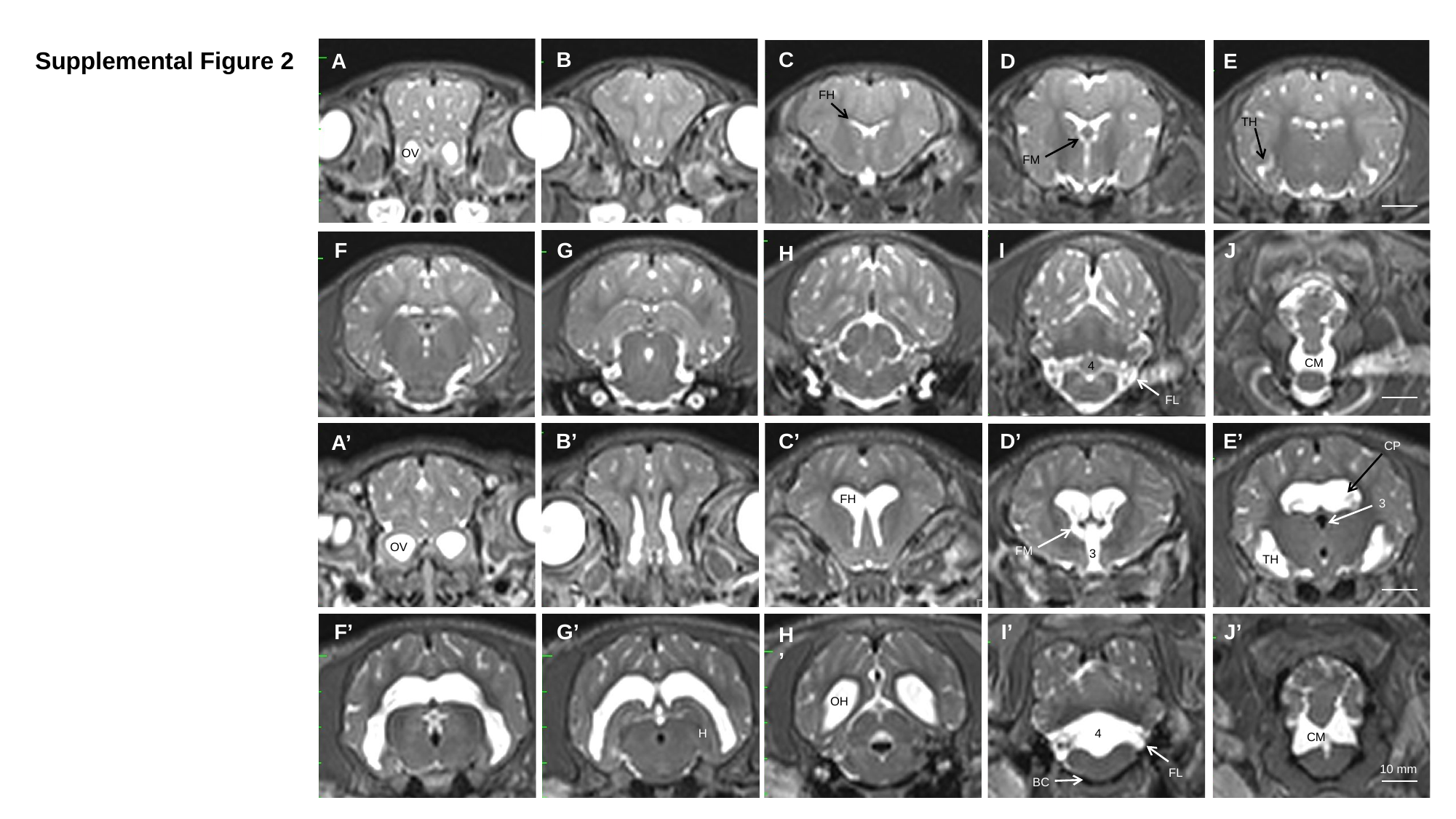

Supplemental Figure 2
C
B
D
E
A
FH
TH
OV
FM
G
I
J
F
H
CM
4
FL
C’
D’
E’
B’
A’
CP
FH
3
OV
FM
3
TH
G’
I’
J’
F’
H’
OH
4
H
CM
10 mm
FL
BC
Supplemental Figure 2: Representative T2-weighted coronal images summarizing brain and ventricular morphology in non-hydrocephalic control (A-J) and hydrocephalic pre-shunt (A’-J’) pigs; panels arranged left to right from anterior to posterior and matched to corresponding levels (i.e. A and A’). Pre-shunt images are taken from case 13 at 16-days post-kaolin. The control pig (case 25) is 28-days old, 2-days before kaolin induction. In the hydrocephalic pre-shunt condition, note the enlargement of all cerebral ventricles and the cisterna magna (CM), prominent flow voids (black) within the third ventricle (3, E’) indicative of high CSF pulsatility, the large olfactory ventricles (OV), and the choroid plexus (CP) in the lateral ventricle LV, and the kaolin (black) blockage of the basal cisterns (BC) and foramina of Luschka (FL). 4 – fourth ventricle, H – hippocampus, FH – frontal horn, FM – foramen of Monro, OH – occipital horn, TH – temporal horn. Scale bar = 10 mm for all panels.
