# Supplementary material for: A novel model of acquired hydrocephalus for evaluation of neurosurgical treatments
Source: Fluids Barriers CNS. 2021 Nov 8;18:49. doi: 10.1186/s12987-021-00281-0 (PMC8576945; doi:10.1186/s12987-021-00281-0)
Supplement: Supplementary file 5 — Additional file 5: Table S1. Medications. [file 12987_2021_281_MOESM5_ESM.docx]

| **Agent** | **Type** | **Dosage** | **Route** | **Frequency** | **Purpose** |
| --- | --- | --- | --- | --- | --- |
| Buprenorphine (Buprenex) | Analgesic | 0.005-  0.02 mg/kg | IM | As needed | Post-operative analgesia |
| Buprenorphine SR (slow release) | Analgesic | 0.12-0.24 mg/kg | SC | Once; duration 3 days | Post-operative analgesia with extended release |
| Rimadyl (Carprofen) | NSAID | 2-4mg/kg | SC | SID for 3 days as needed | Post-operative analgesia and neuroinflammation |
| Cefazolin | Antibiotic | 15-25mg/kg | IM | Q8 1st day post-op;  2^nd^ day as needed | Antibiotic |
| Dexamethasone | Anti-inflammatory | 20mg/kg | IM | Once, as needed;  possibly in combination with Ketamine | Post-operative neuroinflammation |
| Isoflurane | Anesthetic | 1-4% | IH | Continuous for 1-2 Hours | General anesthesia |
| Ketamine | Anesthetic | 1-20mg/kg alone;2.2mg/kg (combined W/ Telazol &  Xylazine) | IM | Once preoperatively | Sedation prior to intubation and reservoir tapping |
| Marcaine | Local Anesthetic | 0.5% | SC | Once as needed | Local anesthetic for skin closure |
| Sodium Pentobarbital | Anesthetic | >120mg/kg, 390mg/ml | IV | Once | Euthanasia while under general anesthesia |
| Xylazine | Analgesic | 2.2mg/kg (combined  With Ketamine) | IM | Once preoperatively | Sedation prior to intubation and maintained anesthesia |
| Levetiracetam  (Keppra) | Anti-seizure | 20mg/kg | PO | BID initially; TID if needed | Seizure control |

**Supplemental Table 1.** Medications

BID - twice/day PO - oral

IM - intramuscular SC - subcutaneous

IV - intravenous SID - once/day

NSAID - non-steroidal anti-inflammatory drug TID - 3 times/day
